# 2121. The Impact of Donor CMV Serostatus on Outcomes of CMV Infections in the Era of Letermovir

**DOI:** 10.1093/ofid/ofac492.1742

**Published:** 2022-12-15

**Authors:** Oscar Morado Aramburo, Amy Spallone, Fareed Khawaja, Joseph Sassine, Krithia Srinivasan, Anthony J Febres-Aldana, Terri Lynn Shigle, Gabriella Rondon, Jeremy Ramdial, Elizabeth Shpall, Ella Ariza-Heredia, Roy F Chemaly

**Affiliations:** The University of Texas MD Anderson Cancer Center, houston, Texas; University of Texas MD Anderson Cancer Center, Houston, Texas; The University of Texas MD Anderson Cancer Center, houston, Texas; University of Oklahoma Health Sciences Center, Oklahoma City, Oklahoma; Stanford, Palo Alto, California; The University of Texas MD Anderson Cancer Center, houston, Texas; The University of Texas MD Anderson Cancer Center, houston, Texas; The University of Texas MD Anderson Cancer Center, houston, Texas; University of Texas MD Anderson Cancer Center, Houston, Texas; The University of Texas MD Anderson Cancer Center, houston, Texas; The University of Texas MD Anderson Cancer Center, houston, Texas; MD Anderson, Houston, Texas

## Abstract

**Background:**

Cytomegalovirus (CMV) infection is a frequent complication after allogeneic hematopoietic cell transplant (allo-HCT) and may increase the risk of other viral infections through its immunomodulatory effects. Donor CMV serology (seropositive [D+] or seronegative donor [D-]) in CMV-seropositive (R+) or seronegative recipients (R-) may impact outcomes post allo-HCT. We analyzed the significance of donor CMV serostatus in a large cohort of alloHCT recipients.

**Methods:**

This is a single-center, retrospective cohort study of 651 allo-HCT recipients cared for at our institution between March 2016 and December 2018. Data on baseline demographics, transplant characteristics, preventive strategies, CMV infection characteristics, and transplant-related outcomes (development of graft versus host disease (GVHD), all-cause mortality, and non-relapse mortality) were collected. A univariate analysis was performed for outcomes of interest using CMV serostatus D-/R- as a control group.

**Results:**

Out of the 651 allo-HCT recipients, 77 were D-/R-, 43 D+/R-, 290 D+/R+, and 241 D-/R+ (table 1). Most patients underwent HCT for AML (40%), received myeloablative conditioning (51%), and had a matched unrelated donor (MUD) HCT (46%). In 2018, letermovir was used in 27% of the D+/R+, 18% of the D-/R+ allo-HCT recipients (table 1) for a total of 116 (55%) allo-HCT recipients. Compared to the CMV D-/R- group, D+/R+ and D-/R+ groups (table 2) had a greater incidence of clinically signicant CMV infections (CS-CMVi) (3.9% vs. 40% vs. 50.6%; all p< 0.01, respectively), CMV end organ disease (0% vs. 14.8% vs. 19.1%; all p< 0.001, respectively), and refractory/resistant (R/R) CMV infections (0% vs. 5.5% vs. 12.4%; all p< 0.03, respectively) with 48 weeks of allo-HCT. CS-CMVi and R/R CMV was more common in D-/R+ allo-HCT when compared to D+/R+ group (50.6% vs. 40.0%, p< 0.001). D-/R+ allo-HCT had worse non-relapse mortality at day 100 compared to D-/R- (3.9% vs. 10.8%, p=0.07).

**Table 1:**
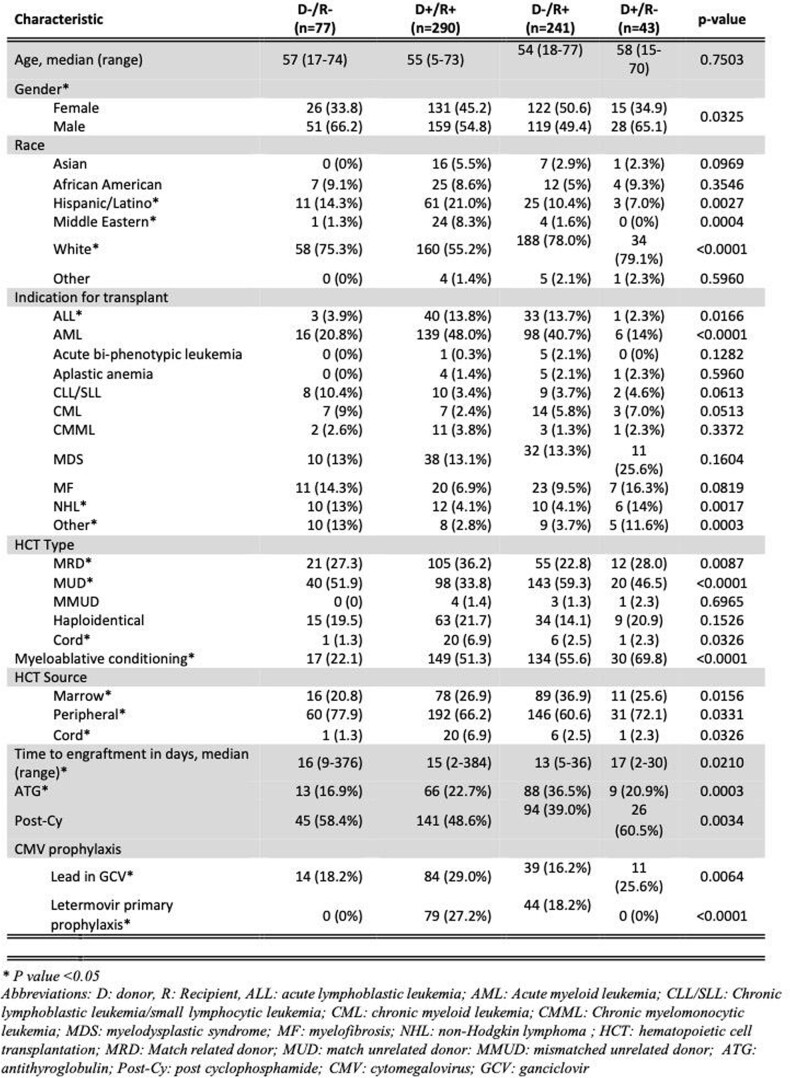
Baseline characteristics

**Table 2:**
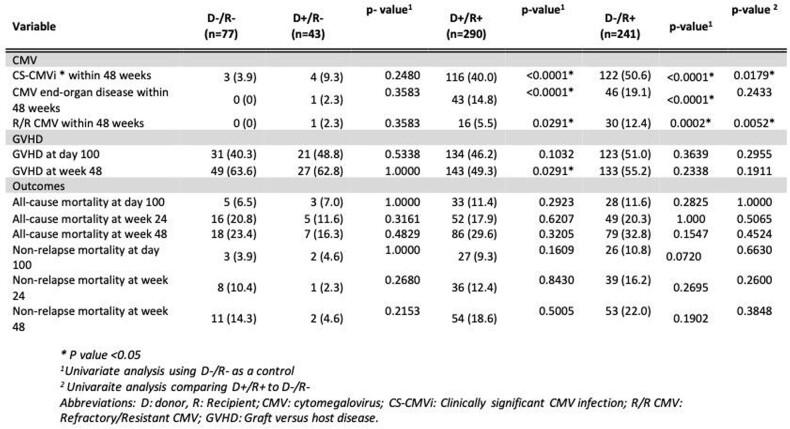
Outcome analysis

**Conclusion:**

Allo-HCT recipients with CMV seronegative donor and recipient had less CMV related complications and a trend towards better survival when compared to D-/R+ allo-HCT. CMV D-/R+ HCT recipients had greater CMV related complications when compared to CMV D+/R+ HCT recipients, possibly due to the protective affect of donor seropositivity.

**Disclosures:**

**Terri Lynn Shigle, PharmD, BCOP**, Takeda: Advisor/Consultant **Gabriella Rondon, MD**, Omeros: Advisor/Consultant **Elizabeth Shpall, MD**, Adaptimmune: Advisor/Consultant|Affimed: License agreement|Axio: Advisor/Consultant|Bayer Helathcare Pharmaceuticals: Honoraria|Fibroblasts and FibrioBiologics: Advisor/Consultant|Navan: Advisor/Consultant|NY Blood Center: Advisor/Consultant|Takeda: License agreement **Ella Ariza-Heredia, MD**, MERCK: Grant/Research Support **Roy F. Chemaly, MD/MPH**, Karius: Advisor/Consultant|Karius: Grant/Research Support.

